# Narrow-band UV-B (TL-01) Phototherapy Exposure and Skin Cancer Risk: A Nested Case-control Study

**DOI:** 10.2340/actadv.v106.adv-2025-0274

**Published:** 2026-09-09

**Authors:** Petra Åkerla, Eero Pukkala, Mika Helminen, Niina Korhonen, Toni Karppinen

**Affiliations:** 1 Department of Dermatology, Tampere University Hospital, Wellbeing Services County of Pirkanmaa, Tampere, Finland; 2 Faculty of Medicine and Health Technology, Tampere University, Tampere, Finland; 3 Health Sciences Unit, Faculty of Social Sciences, Tampere University, Tampere, Finland; 4 Finnish Cancer Registry, Institute for Statistical and Epidemiological Cancer Research, Helsinki, Finland; 5 Tays Research Services, Tampere University Hospital, Wellbeing Services County of Pirkanmaa, Tampere, Finland

**Keywords:** basal cell carcinoma, keratinocyte cancer, narrow band UVB radiation, photocarcinogenicity, phototherapy, skin cancer risk

## Abstract

Narrow-band TL-01 ultraviolet B phototherapy (TL-01) is generally considered a relatively safe treatment for skin diseases, although conflicting findings have also been reported. This study aimed to evaluate the association between TL-01 exposure and skin cancer risk using a nested case-control design within a Finnish cohort of 4,815 patients treated with TL-01 monotherapy, with subsequent 38 cases of basal cell carcinoma, 13 cases of cutaneous melanoma, and 9 cases of cutaneous squamous cell carcinoma. Four matched controls were selected for each skin cancer case. Personal TL-01 exposure data were obtained from hospital records. Conditional logistic regression was used to estimate odds ratios (ORs) and 95 % confidence intervals (CIs). An increased risk for basal cell carcinoma was observed with higher TL-01 exposure (OR per 20 sessions 1.36, 95% CI 1.08–1.72), whereas no association was found for cutaneous melanoma or cutaneous squamous cell carcinoma. In a categorical analysis, with category of 1–29 TL-01 sessions as the reference, the risk of basal cell carcinoma was markedly elevated among patients who received ≥60 sessions (OR 4.16, 95% CI 1.49–11.6). Occupational outdoor work was considered a potential confounder, but it had no effect. Further studies with larger cohorts and longer follow-up are needed to confirm these findings.

**SIGNIFICANCE**

Narrow-band TL-01 UVB phototherapy is widely used to treat common skin diseases. However, its long-term effects on skin cancer risk are not fully understood. In this Finnish study among patients treated with TL-01 UVB phototherapy, those who developed basal cell carcinoma, the most common type of skin cancer, had received more TL-01 treatment sessions than those without basal cell carcinoma. A similar difference was not found for melanoma or cutaneous squamous cell carcinoma. These findings highlight the importance of monitoring the cumulative TL-01 exposure and support further research to examine the long-term safety of TL-01 phototherapy.

Phototherapy is a widely used treatment modality for many skin diseases, including psoriasis and atopic dermatitis. For several years, phototherapy has typically been administered using the Philips ultraviolet B narrowband TL 100W/01 lamp, emitting narrowband ultraviolet B (UVB) light at 311–313 nm. This treatment is known as narrow-band TL-01 ultraviolet B phototherapy (TL-01 phototherapy). In the 1980s, it was shown that these wavelengths had the most effective therapeutic impact on psoriasis (1, 2). Since then, TL-01 phototherapy has gradually replaced other modalities, e.g. psoralen-ultraviolet A photochemotherapy (PUVA) and broadband UVB treatment, becoming the primary choice for phototherapy in recent decades.

Solar ultraviolet (UV) radiation is known to be a major risk factor for skin cancers and UV radiation, including UVB, is classified as a Group 1 carcinogen to humans (3, 4). Photocarcinogenic risk of PUVA-therapy has previously been established and the main concern with high doses of PUVA is the increased risk of both keratinocyte cancer (basal cell carcinoma [BCC], cutaneous squamous cell carcinoma [cSCC]) and cutaneous melanoma (CM) (5–8). Instead, TL-01 phototherapy has been considered a relatively safe treatment option for skin diseases due to its presumed more favourable long-term risk profile. No firm association between TL-01 treatment and increased skin cancer risk has been shown among patients with psoriasis or atopic dermatitis (9–11). Studies though tend to have some limitations concerning sample sizes, follow-up times and confounding factors such as other sources of UV exposure. Hence, the photocarcinogenicity of TL-01 therapy remains a concern to some extent, and further study is needed to better understand its potential carcinogenic effects.

Our recent multicentre study, which investigated the risk of skin cancer among patients with psoriasis and atopic dermatitis treated with TL-01 monotherapy in Finland, revealed an increased incidence of BCC, cSCC and CM in the study population compared to the respective incidence rates in the general population (12). The aim of this study was to assess the role of TL-01 exposure in the observed increase in skin cancer incidence within a nested case-control design.

## MATERIALS AND METHODS

### Study participants

We conducted a case-control study nested within a cohort of 4,815 patients with psoriasis or atopic dermatitis treated with TL-01 phototherapy in Finland during 1999–2019. These patients did not receive any other phototherapy modalities (e.g. PUVA treatment). The detailed methods of collecting the cohort have been described previously (12). The cohort consists of 2,443 women and 2,372 men, with 54% of patients treated for psoriasis and 4646 for atopic dermatitis. The incident skin cancer cases diagnosed between 1999 and 2020 among the cohort patients were identified from the Finnish Cancer Registry (FCR). The FCR has collected information on cancer cases diagnosed since 1953. Data are received from several sources such as hospitals, private physicians, pathological laboratories and death certificates (13). Out of skin cancers, only histologically verified ones are registered. The 3 most common types of skin cancers, (i.e.) BCC, cSCC and CM, were selected for analysis in this study.

Four controls for each case (i.e. patient with skin cancer) were randomly selected from the cohort. They were matched by sex, skin disease (psoriasis or atopic dermatitis) and year of birth (±2 years). The controls had to be alive at the time of the cancer diagnosis of the respective case (“index date”) and had to have TL-01 phototherapy starting before the index date. Four controls were found for every CM case, but for one cSCC case only 3 eligible controls were available. For the BCC cases, 3 controls were found for one case and only one control for another case. The final case-control groups were as follows: 38 cases of BCC and 148 controls, 13 cases of CM and 52 controls and 9 cases of cSCC and 35 controls.

### Exposure assessments

The exposure to TL-01 phototherapy for each case and control was examined by collecting personal phototherapy details (the first TL-01 administration, total number and duration of therapeutic sessions) from the patient phototherapy registers of 17 Finnish Central and University Hospitals. Only TL-01 exposure before the index date was considered.

As a potential confounder we also assessed the occupational outdoor work exposure of cases and controls based on job history information of the participants obtained from Statistics Finland. The classification of occupation is based on the International Standard Classification of Occupation 2008 (ISCO-08). The occupational categories were classified as outdoor and indoor workers using information of the Finnish job-exposure matrix (FINJEM) (14). Every person who has ever had an outdoor occupation was classified in the group of outdoor workers, and all others in the group of indoor workers. Outdoor occupations included farm and forestry workers, timbermen, painters, gardeners, bricklayers, asphalt workers, building hands, chimney sweeps, stevedores, engineer surveyors and cartographers, biologists, shipmasters, physical education instructors and trainers, photographers, directors and nursing staff at child day care centres, and postal workers.

### Statistical analysis

Conditional logistic regression was used to generate odds ratios (ORs) and 95% confidence intervals (CIs). Skin cancer risk was the dependent variable. TL-01 phototherapy and occupation (outdoor or indoor work) were used as independent variables.

The effect of the number of TL-01 sessions was analysed both as a continuous variable and in 3 categories. The categories were based on the convention that a typical TL-01 treatment period consists of 15–20 sessions and rarely exceeds 25 sessions. The reference category (1–29 TL-01 sessions) was intended to reliably include all patients who had received only a single course of TL-01 phototherapy; therefore, the upper limit was set at 29 sessions rather than 20. Consequently, the reference category represents patients who received, on average, 1–2 phototherapy courses, the middle category (30–59 sessions) includes those with intermediate exposure, and the highest category (≥60 sessions) comprises patients who underwent at least 3–4 treatment courses.

All analyses were conducted by using R statistical software version 4.3.2 (R Core Team, 2023; R Foundation for Statistical Computing, Vienna, Austria) and RStudio (Posit Team, 2024). A 2-sided *p*-value < 0.05 was defined as statistically significant.

## RESULTS

Of the 60 cases, 61.7% were males and 38.3% (**Table I**). The mean age at skin cancer diagnosis was 68 years for BCC, 60 years for CM, and 72 years for cSCC (with median ages of 70, 62, and 73 years, respectively). The median time from the beginning of phototherapy to the index date was 6.1 years for BCC (range: 9 days to 19.8 years), 2.8 years for CM (2.4 months to 11.9 years) and 5.3 years for cSCC (11.5 months to 11.4 years).

**Table I.** Selected demographic characteristics of skin cancer cases and controls

	Basal cell carcinoma		Cutaneous melanoma		Cutaneous squamous cell carcinoma	
Characteristics	Cases (*n*=38)	Controls (*n*=148)	Cases (*n*=13)	Controls (*n*=52)	Cases (*n*=9)	Controls (*n*=35)
Sex *n* (%)						
Female	13 (34.2)	52 (35.1)	4 (30.8)	16 (30.8)	6 (66.7)	23 (65.7)
Male	25 (65.8)	96 (64.9)	9 (69.2)	36 (69.2)	3 (33.3)	12 (34.3)
Age at index date, years, *n* (%)^a^						
≤40 years	0 (0)	0 (0)	1 (7.7)	6 (11.5)	0 (0)	0 (0)
41–60 years	8 (21.1)	34 (23.0)	5 (38.5)	19 (36.5)	2 (22.2)	8 (22.9)
60–80 years	28 (73.7)	108 (73.0)	5 (38.4)	19 (36.5)	5 (55.6)	20 (57.1)
>80 years	2 (5.3)	6 (4.1)	2 (15.4)	8 (15.4)	2 (22.2)	7 (20.0)
Year of birth, *n* (%)						
≤1,940	4 (10.5)	13 (8.8)	2 (15.4)	8 (15.4)	4 (44.4)	11 (31.4)
1941–1960	29 (76.3)	115 (77.7)	6 (46.2)	22 (42.3)	3 (33.3)	19 (54.3)
1961–1980	5 (13.2)	20 (13.5)	5 (38.5)	22 (42.3)	2 (22.2)	5 (14.3)
Skin disease, *n* (%)						
Atopic dermatitis	3 (7.9)	9 (6.1)	3 (23.1)	12 (23.1)	4 (44.4)	16 (45.7)
Psoriasis	35 (92.1)	139 (93.9)	10 (76.9)	40 (76.9)	5 (55.6)	19 (54.3)
TL-01 sessions received						
Median (mean)	29 (44.7)	20 (28.6)	17 (19.2)	20 (31.5)	20 (23.1)	16 (25.5)
Range	4–215	4–161	6–51	7–215	8–48	4–138
Occupation, *n* (%)						
Indoor	33 (86.8)	127 (85.8)	13 (100)	41 (78.8)	9 (100)	33 (94.3)
Outdoor	5 (13.2)	21 (14.2)	0 (0)	11 (21.1)	0 (0)	2 (5.7)

^a^Date of skin cancer diagnosis in the case-control set.

The ORs for TL-01 treatment were not elevated for CM or cSCC, while OR was statistically significantly elevated for BCC (OR for one TL-01 treatment=1.016, 95% CI 1.004–1.027). To demonstrate the practical relevance of the results, we calculated the OR for BCC per 20 sessions (the average number of TL-01 therapeutic sessions received per treatment period), which was 1.36 (95% CI 1.08–1.72) (**Table II**). Patients with BCC had been exposed to higher number of TL-01 treatment sessions than their controls. The BCC cases received a median of 29 TL-01 treatment sessions (mean 45, range 4–215) whereas the controls were treated a median of 20 sessions (mean 29, range 4–161).

**Table II.** Odds ratios (ORs) and 95 % confidence intervals (CIs) for TL-01 phototherapy exposure and different skin cancers

Skin cancer type	Cases	Controls	Per one TL-session		Per 20 TL -sessions	
	*n*	*n*	OR	95% CI	OR	95% CI
Basal cell carcinoma	38	148	1.016	1.004–1.027	1.36	1.08–1.72
Cutaneous melanoma	13	52	0.962	0.908–1.020	0.46	0.14–1.48
Cutaneous squamous cell carcinoma	9	35	0.997	0.967–1.028	0.94	0.51–1.73

A sensitivity analysis excluding skin cancer cases diagnosed within the first year after the latest TL-01 phototherapy (*n*=7), along with their matched controls (*n*=27) and any additional controls with less than 1 year between their last phototherapy and the index date (*n*=1), was also conducted. The results did not change substantially: for BCC, the OR per 20 TL-01 sessions was 1.46 (95% CI 1.13–1.88), and for CM and cSCC, the ORs remained nonelevated (Table SI).

**Fig. 1.**
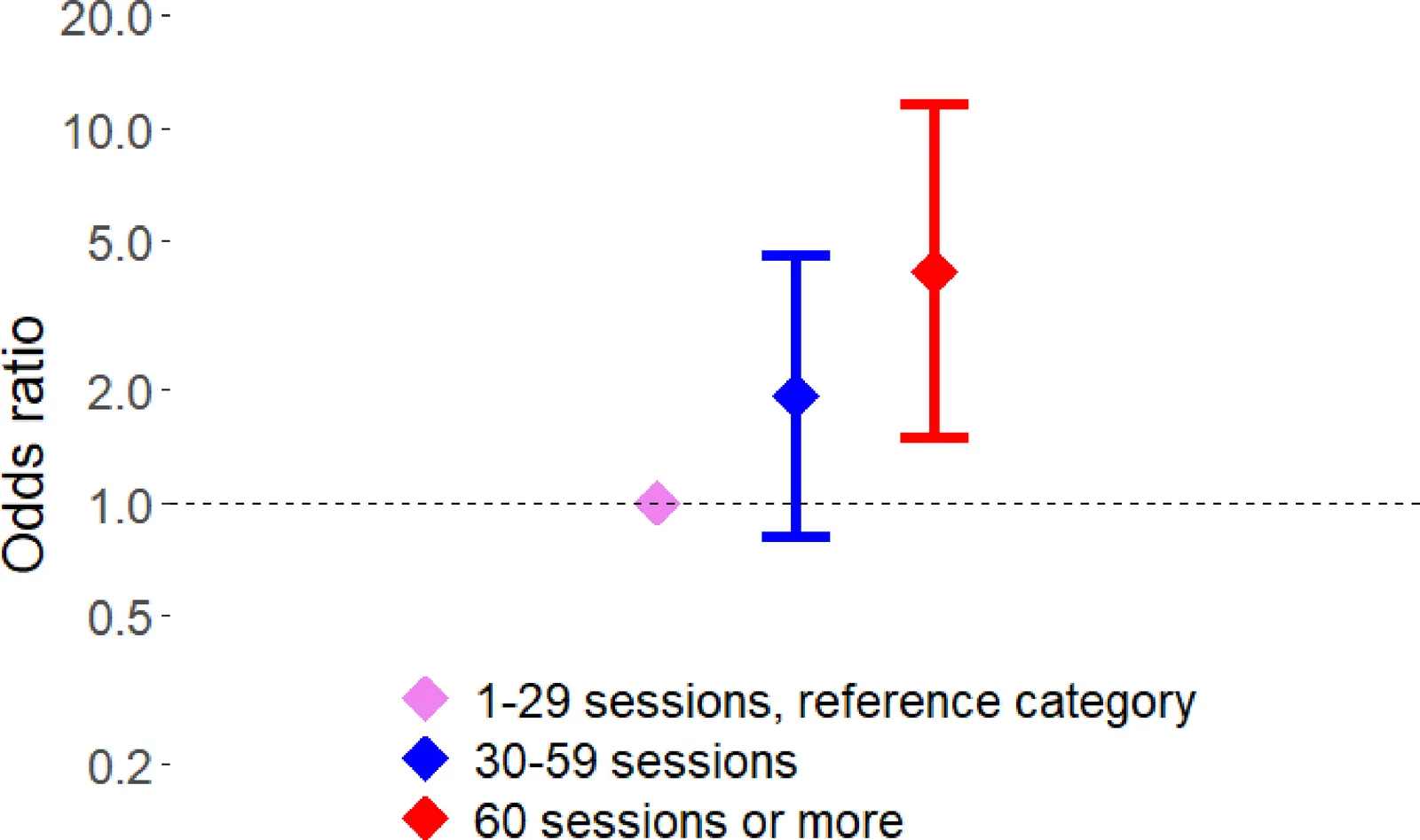
Odds ratios with 95 % confidence interval bars for basal cell carcinoma, by number of TL-01 sessions received.

In a categorical analysis, with category of 1–29 TL-01 sessions as the reference, the risk was markedly elevated among those who received ≥60 sessions (OR 4.16, 95% CI 1.49–11.6) and less elevated among those who received 30–59 sessions (OR 1.92, 95% CI 0.81–4.60) (**Fig. 1**). The mean number of TL-01 sessions within each category was 16.8 in the reference group, 42.5 in the middle category, and 102.7 in the ≥60 sessions group.

Adjustment for outdoor work did not change the results. There was no association between outdoor work and the elevated risk of BCC in our data (adjusted OR for one TL-01 treatment=1.016, 95% CI 1.004–1.028). None of the CM or cSCC cases had a history of outdoor work.

## DISCUSSION

The current study showed an association between higher number of TL-01 phototherapy sessions and BCC risk but did not suggest an association concerning cSCC or CM risk. The increased risk for BCC was not dependent on occupational UV exposure.

There are only a few previous studies concerning the carcinogenicity and dose-dependent effects of TL-01 monotherapy. A recent Canadian cohort study involving 2650 TL-01 -treated patients (some also treated with broad-band UVB), most of whom had psoriasis or eczema, observed 29 skin cancer cases and found no dose-response correlation between the numbers of treatment or cumulative UV doses and skin cancer risk (15). Another cohort study from Israel with 767 TL-01 treated patients, mostly with psoriasis, and of whom 34 developed skin tumours, did not find a difference in the mean number of TL-01 treatments among patients who developed skin cancer and those who did not (16). In a Taiwanese cohort study involving 1241 patients with atopic dermatitis with a history of UV-B phototherapy, out of whom 6 developed skin cancers, there was no association between the risk of skin cancers and the number of phototherapy sessions (10).

In the current study, both cases and controls were derived from a cohort of TL-01 treated patients receiving regular dermatological follow-up, rather than being compared with the general population, who typically lack routine dermatologist surveillance. Given that skin cancer development, particularly that of BCC, generally occurs over a prolonged period, diagnostic delays between the appearance of a suspicious skin lesion and a confirmed cancer diagnosis can be substantial in the general population. Because both cases and controls in our study were under regular dermatological follow-up, it can be assumed that any such diagnostic delays were comparable between the groups, thereby minimizing the potential for detection bias. In Finland, phototherapy procedures are largely standardized, and treatment sessions are typically conducted under the supervision of nurses unless specific concerns arise. Follow-up visits with dermatologists, including comprehensive skin examinations, are conducted separately. Therefore, higher TL-01 exposure does not automatically result in more frequent clinical examinations, and cases and controls can be assumed to have been in a similar position with respect to dermatological surveillance. Our study population was also carefully selected to exclude patients who had ever received other phototherapy modalities, such as PUVA and broad-band UVB, which makes it possible to examine the photocarcinogenic role of TL-01 as a monotherapy.

We considered the work histories of the study participants to identify the potential confounding effects of occupational UV exposure. A recent review of 32 epidemiological studies reported that outdoor workers have a 20% increase in risk of developing BCC compared with indoor workers. However, this association largely disappeared in studies with a low risk of bias, indicating that regular outdoor work may not substantially elevate BCC risk (17). A large Nordic cohort study, including over 14 million study subjects, showed that the risk of CM is increased among indoor workers (standardized incidence ratio [SIR] 1.09) and decreased among outdoor workers (SIR 0.79) when comparing to the general population (18). In the case of CM, particularly intermittent and intense UV exposure, as well as sunburns, are known to increase skin cancer risk, suggesting that nonoccupational, recreational UV exposure plays a critical role in its development. For cSCC, cumulative exposure to solar UV radiation is known to be a major risk factor (19). A meta-analysis of 18 studies from Europe, North America and Australia found a positive association between occupational UV exposure and an increased risk of cSCC among outdoor workers (20). A large Nordic study also indicated that cSCC risk was associated to some extent with occupational UV exposure (21). In our Finnish data, occupational status did not appear to affect the risk of BCC. However, it must be noted that the number of outdoor workers in our dataset was limited, and such a rare and weak factor would not be expected to have a notable impact on the results.

Due to the low number of skin cancer cases in our study, it was not reasonable to analyse patients with atopic dermatitis and psoriasis separately. Both cases and controls also had relatively low levels of TL-01 exposure, and the difference in the number of TL-01 sessions received between the cases and controls was small. Still, we observed a dose-response trend by the number of sessions received, with a more than 4-fold BCC risk among those exposed to more than 60 TL-01 sessions (approximately 3–4 treatment periods) as compared to those with less than 30 sessions. A larger cohort with greater TL-01 exposure would have enhanced the applicability of our results to practical clinical settings. The number of cSCC and CM cases in our dataset was low; therefore, our results do not allow for reliable conclusions about the association between these skin cancers and TL-01 phototherapy.

Since it can take several years for skin cancer to develop, recent UV exposure is unlikely to be related to cancer risk. Within this study, we were unable to account for an appropriate lag period in the initiation phase, and TL-01 phototherapy is rather considered as a potential moderating factor in the process of developing skin cancer. Another limitation of our registry-based study is the absence of information on potential confounding factors other than outdoor work, such as sunbathing habits, sun vacations, skin phototype and long-term use of systemic immunosuppressive medications. Additionally, data on the doses administered per individual session were insufficiently available. Fortunately, established TL-01 phototherapy protocols issued by the Finnish Dermatological Society, including guidelines on treatment initiation and dose increments, are generally used across phototherapy units in Finland, making TL-01 dosing relatively consistent.

In conclusion, our study suggests that among TL-01 treated patients the number of phototherapy sessions did not correlate to developing cSCC or CM but increasing exposure to TL-01 phototherapy was associated with BCC risk. Further studies involving larger cohorts with TL-01-treated patients and longer follow-up periods are needed to confirm these findings.

## Data Availability

The data are not publicly available due to privacy and ethical restrictions, as the research approval does not permit data sharing.

## Supplementary material

Table SI.
