# Supplementary material for: Narrow-band UV-B (TL-01) Phototherapy Exposure and Skin Cancer Risk: A Nested Case-control Study
Source: Acta Derm Venereol. 2026 Sep 9;106:0274. doi: 10.2340/actadv.v106.adv-2025-0274 (PMC13555599; doi:10.2340/actadv.v106.adv-2025-0274)
Supplement: Supplementary file 1 [file ActaDv-106-0274-s0001.pdf]

Table SI. Odds ratios (ORs) and 95% confidence intervals (CIs) for the association between TL-01 phototherapy exposure and skin cancer risk in a one-year lag-time sensitivity analysis.

Skin cancer type	Cases	Controls	Per one TL-session		Per 20 TL -sessions	
	n	*n*	OR	95% CI	OR	95% CI
Basal cell carcinoma	35	136	1.019	1.006-1.032	1.46	1.13-1.88
Cutaneous melanoma	10	40	0.970	0.917-1.022	0.54	0.19-1.55
Cutaneous squamous cell carcinoma	8	31	0.994	0.964-1.025	0.89	0.48-1.65
